# Enhancing Neonatal Care: The Vital Role of Pulse Oximetry in the Early Screening of Critical Congenital Heart Diseases and Respiratory Diseases in Rural Areas

**DOI:** 10.7759/cureus.58398

**Published:** 2024-04-16

**Authors:** Prajyoth M Gaonkar, Saurabh R Mutha, Isha M Sanghani

**Affiliations:** 1 Department of Pediatrics, Punyashlok Ahilyadevi Holkar Government Medical College, Baramati, IND

**Keywords:** congenital heart disease (chd), pulse oximetry screening, persistent pulmonary hypertension, respiratory distress syndrome (rds), critical congenital heart disease

## Abstract

Background

Pulse oximetry screening (POS) is acknowledged globally as a noninvasive method to detect critical congenital heart diseases (CCHDs) and respiratory illnesses. However, its value for early diagnosis and treatment remains unrecognized in many hospitals with limited resources around the world. This study aimed to evaluate POS’s application in CCHDs, persistent pulmonary hypertension (PPHN), and respiratory distress syndrome (RDS) for early diagnosis and its influence on clinical procedures in rural areas.

Methods

This prospective observational study included all eligible newborn infants in the regional neonatal unit of a community healthcare center. Their peripheral oxygen saturation was assessed at <24 hours and >24 hours after birth, in the right upper limb and either lower limb. An oxygen saturation of <95% or >3% difference between pre-ductal and post-ductal circulations was considered abnormal. All neonates with abnormal oxygen saturations at >24 hours after birth were subjected to another POS test within two hours of the last test. If the oxygen saturation was still abnormal, it was considered a positive POS test. The POS results were classified as oxygen saturation abnormal (<90%), abnormal (90-94%), and normal (≥95%). All neonates with a positive POS test were referred for echocardiography.

Results

Overall, 440 infants had documented POS results. A total of 65 (14.77%) infants had a positive POS test result, out of which 39 (8.86%) cases were diagnosed on further evaluation. Four neonates had CCHD (positive predictive value (PPV) = 6.15%), 26 had RDS (PPV = 40%), and nine had PPHN (PPV = 13.85%). Without any further delay, the doctor directed them all to a more advanced facility.

Conclusion

Our research showed that, in large-scale clinical settings, the addition of pulse oximetry to routine cardiac auscultation could be a reliable and feasible method to screen newborns for CCHD, PPHN, and RDS early on. Our research underscores the importance of implementing routine POS to detect CCHD, RDS, and PPHN in clinical practice.

## Introduction

Pulse oximetry is extensively utilized in critical care settings due to its ability to promptly detect severe hypoxemia, enabling timely intervention and mitigating potential risks [1]. The application of pulse oximetry in neonatal care has expanded over recent years, with a growing body of contemporary research highlighting its relevance and limitations. Numerous studies have explored its efficacy in detecting critical congenital heart diseases (CCHDs) [2], its role in assessing preterm infants’ respiratory status [3], and its use in guiding oxygen therapy for neonates with respiratory distress [4].

Globally, neonatal mortality is predominantly attributed to congenital heart disease, sepsis, and lower respiratory tract infections [5]. The incidence of congenital heart defects (CHDs) stands at approximately seven to nine per 1,000 live births [6,7]. Neonates are routinely screened with pulse oximetry in various countries across the world [8]. CHDs account for nearly half of all deaths resulting from congenital anomalies and up to 10% of all infant fatalities in Western countries [8,9]. Mortality rates associated with CHDs range from 3% to 7% in industrialized nations to as high as 20% in developing regions [4]. Critical CHD results in death within the first month after birth [10]. Timely diagnosis and intervention can often correct most of these defects, while late detection can lead to complications, including acute cardiovascular collapse upon closing of the duct-dependent circulation. Additionally, poor clinical conditions at the time of surgery can exacerbate outcomes and increase mortality [11,12]. There are discrepancies in the current screening protocols for CHDs, and many newborns with CCHD go undetected before being released from the hospital [13,14]. Antenatal ultrasound screening at 20 weeks of pregnancy, aimed at fetal anomaly detection, can identify only approximately 50% [15,16]. Postnatal clinical examinations, involving assessments of heart sounds and visible cyanosis, are similarly inadequate, detecting only 31% of critical CHDs [17-19]. The incorporation of pulse oximetry screening (POS) can significantly enhance the detection of these distresses (CCHD, persistent pulmonary hypertension (PPHN), and respiratory distress syndrome (RDS)), increasing the rate to around 75-90% [17,18]. Prior studies have demonstrated that POS is accurate, cost-effective, and well received by both parents and healthcare staff [8,17-19].

RDS is a common and life-threatening condition in neonates, particularly premature infants. It results from a deficiency of pulmonary surfactant, which leads to inadequate lung expansion and oxygen exchange. RDS predominantly affects premature infants, with incidence rates varying according to the gestational age at birth. It is particularly prevalent in infants born before 28 weeks of gestation. The diagnosis of RDS typically involves clinical assessment, radiographic imaging (such as chest X-rays), and arterial blood gas analysis to assess oxygenation and acid-base status [20]. Treatment for RDS often includes providing supplemental oxygen and administering exogenous surfactant to improve lung function. Mechanical ventilation may be necessary in severe cases [4].

PPHN in newborns is a complex and potentially life-threatening condition characterized by elevated pulmonary vascular resistance, resulting in impaired blood flow to the lungs and inadequate oxygenation. PPHN is relatively rare but can occur in both term and preterm infants. The incidence can vary, and it often arises as a secondary condition to other neonatal issues [21]. The diagnosis of PPHN involves clinical assessment, echocardiography to evaluate pulmonary pressures, and monitoring of oxygenation and blood gases [22]. Treatment strategies may include supplemental oxygen, mechanical ventilation, inhaled nitric oxide therapy, and extracorporeal membrane oxygenation in severe cases [21].

This study meticulously outlines three distinct medical conditions in a neonatal population of 440, detailing the prevalence and characteristics of CCHD, RDS, and PPHN, and is dedicated to assessing the accuracy of POS and its implications for clinical practice, encompassing an evaluation of CCHD, RDS, and PPHN diagnosed after birth that POS did not initially detect.

## Materials and methods

The current investigation was carried out at Punyashlok Ahilyadevi Holkar Government Medical College (PAHGMC), Baramati, India, in the Department of Pediatrics. This was a prospective observational study involving all 440 newborn infants born and admitted to the postnatal ward or NICU in Women Hospital, Baramati, India (a community healthcare center) between September 2023 and February 2024. The data was retrospectively analyzed.

Inclusion criteria and exclusion criteria

All the newborn infants born in the hospital whose parents consented to pulse oximetry and echocardiography (if necessary) were included. Neonates whose parents refused to give consent and those whose prenatal sonography detected CHD before birth were excluded. Moderate to late preterm (32-37 weeks) neonates were included. Others (<32 weeks) were excluded as they were extremely unstable at birth. All the post-term neonates were included.

Study tool

**Figure 1:**
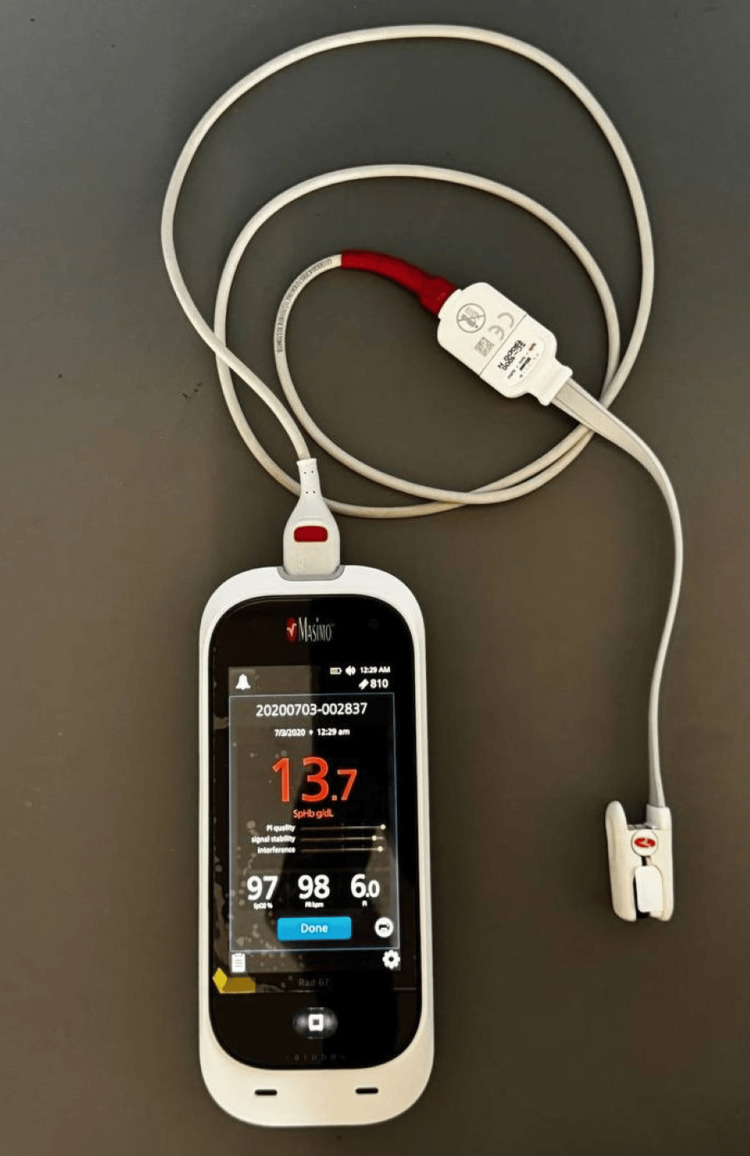
Study tool (Masimo Rad-67® Pulse CO-Oximeter)

The pulse oximeter used for this study was the Masimo Rad-67® Pulse CO-Oximeter (Masimo Corporation, Irvine, California, United States; Figure 1). It is a noninvasive handheld device intended to measure functional oxygen saturation of arterial hemoglobin (SpO2), pulse rate, and perfusion index, along with an optional noninvasive measurement of total hemoglobin (SpHb).

The general principle of a pulse oximeter is to determine blood oxygen saturation by measuring the amount of light absorbed by the oxygenated and deoxygenated hemoglobin in the blood under illumination by red and infrared light. The general principle of a pulse oximeter is to determine blood oxygen saturation by measuring the amount of light absorbed by the oxygenated and deoxygenated hemoglobin in the blood under illumination by red and infrared light [1].

Methodology

**Figure 2:**
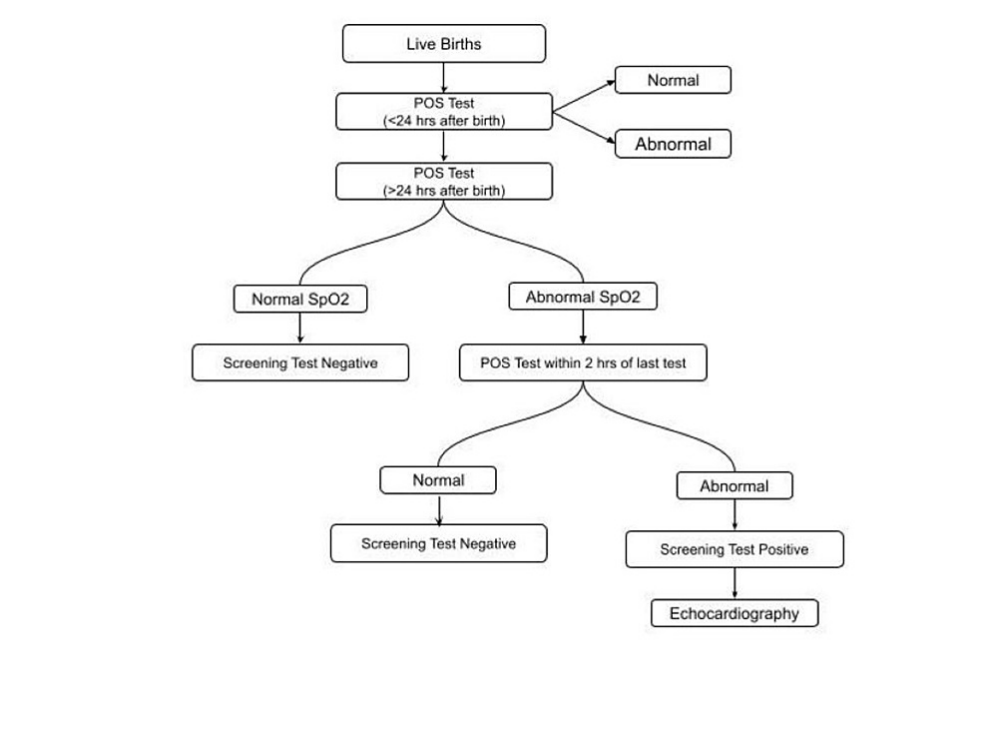
Steps of screening

Parents received a pre-drafted written informed consent form. The screening was routinely conducted by researchers in the postnatal ward, or NICU, following the steps shown in Figure 2. All babies were screened twice, at <24 hours and >24 hours after birth. Records were taken for at least two minutes. Pulse oximeter oxygen saturation (SpO2) was measured from the right hand (pre-ductal) and on either foot (post-ductal). SpO2 levels were recorded on the neonatal registration sheet.

A saturation of ≥95% was classified as a screening test negative. A reading of <95% in either the pre- or post-ductal circulations or a difference of more than 3% between pre- and post-ductal oxygen saturations was considered abnormal. All neonates with an abnormal reading on the second screening >24 hours after birth were subjected to another POS test within two hours of the last test. If the oxygen saturation was still abnormal, it was classified as a screening test positive. All newborns who tested positive on the screening test were referred for a two-dimensional echocardiography.

## Results

We analyzed 440 neonatal individuals in the present study. It is organized based on gender, delivery method, weight categories, and hemoglobin levels (Table 1), while Table 2 presents a comprehensive overview of oxygen saturation levels (SpO2) at different times and ductal locations.

**Table 1:** Presentation of demographic characteristics of neonates Hb, hemoglobin

	Gender		Delivery		Weight (kg)			Hb (g/dL)		
Gestational age	Male	Female	Natural	Cesarean	Below 2.5 kg	2.5-4 kg	Above 4 kg	Below 14	14-18	Above 18
Pre-term	34	28	30	32	43	19	0	19	35	8
Term	196	180	237	139	33	340	3	72	243	61
Post-term	1	1	1	1	1	1	0	0	2	0
Grand total	231	209	268	172	77	360	3	91	280	69
	N = 440									

**Table 2:** Oxygen saturation levels (SpO2) at different time points in pre-ductal and post-ductal circulation The data is categorized into three SpO2 levels: abnormal (<90%), abnormal (90-94%), and normal (≥95%).

SpO2 level	<24 hours pre-ductal	<24 hours post-ductal	>24 hours pre-ductal	>24 hours post-ductal	Within two hours last check pre-ductal	Within two hours last check post-ductal
Abnormal (below 90%)	15 (3.4%)	17 (3.9%)	12 (2.7%)	19 (4.3%)	9 (2.0%)	13 (3.0%)
Abnormal (90-94%)	48 (10.9%)	57( 13.0%)	37 (8.4%)	47 (10.7%)	36 (8.2%)	42 (9.5%)
Normal (above 95%)	377 (85.7%)	366 (83.2%)	391 (88.9%)	374 (85.0%)	33 (7.5%)	23 (5.2%)
N = 440					Done for neonates with abnormal results >24 hours after birth	

**Figure 3:**
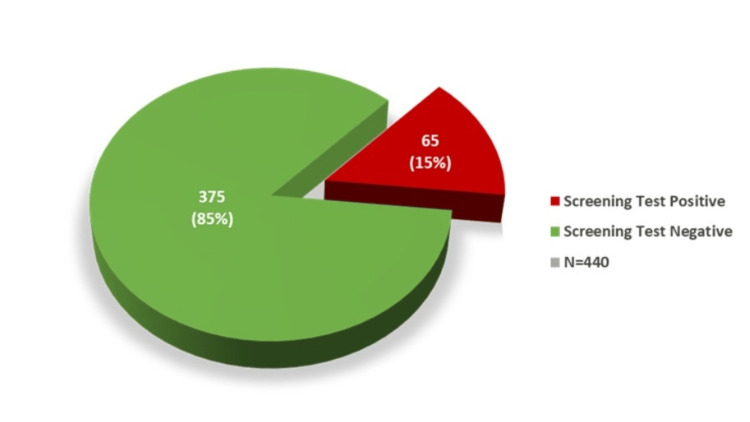
Results of the POS test POS, pulse oximetry screening

**Figure 4:**
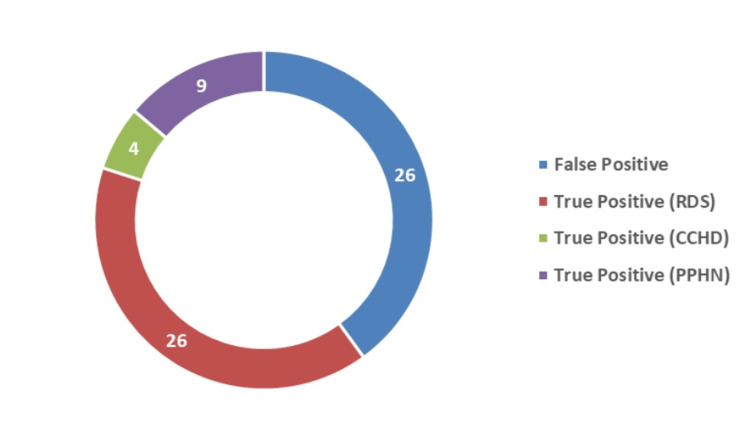
True and false positive cases CCHD, critical congenital heart disease; PPHN, persistent pulmonary hypertension; RDS, respiratory distress syndrome

**Figure 5:**
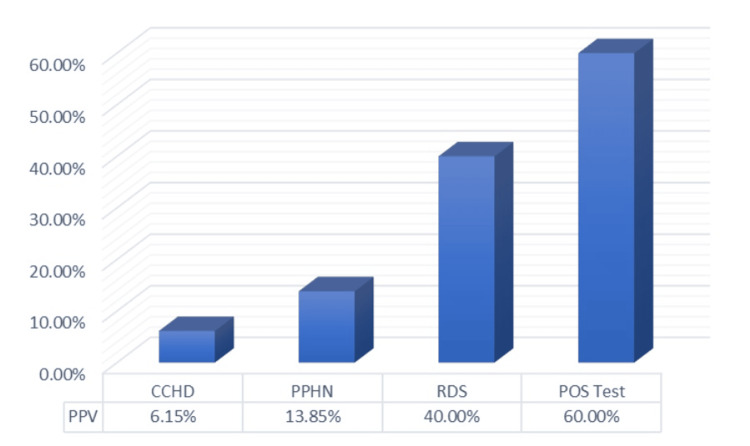
PPV for diagnosed cases CCHD, critical congenital heart disease; PPHN, persistent pulmonary hypertension; PPV, positive predictive value; RDS, respiratory distress syndrome

After the test, 65 neonates (15%) had a screening test-positive result, and 375 neonates (85%) had a negative result (Figure 3). Among the cases that were screening test positive, 39 cases were diagnosed with a disease, making them the true positive cases, while the remaining 26 cases were false positive, giving a positive predictive value (PPV) of 60% for all diagnosed cases (Figures 4, 5). Among the true positive cases, four neonates were diagnosed with critical CHD (PPV = 6.15%), nine had a diagnosis of PPHN (PPV = 13.85%), and 26 had an RDS diagnosis (PPV = 40%), as shown in Figure 4.

**Figure 6:**
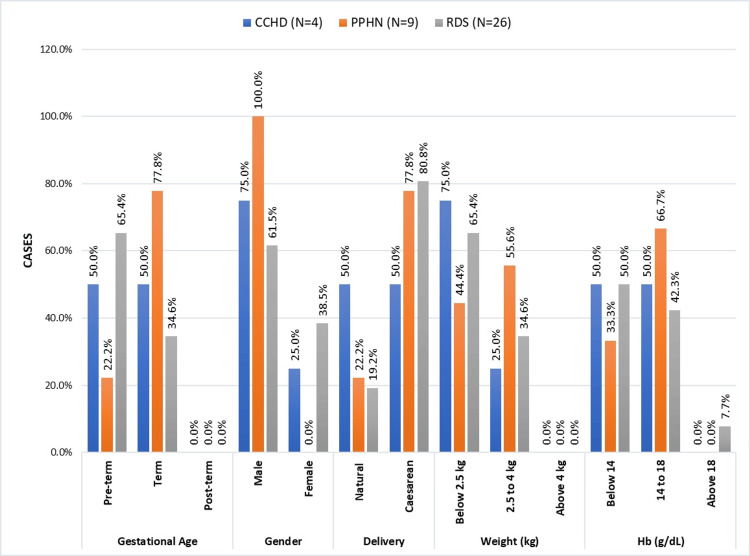
Perinatal factors and physiological measures in CCHD, PPHN, and RDS in infants CCHD, critical congenital heart disease; PPHN, persistent pulmonary hypertension; RDS, respiratory distress syndrome

A comparison of the different perinatal variables and physiological measurements in newborns diagnosed with CCHD, PPHN, and RDS is shown in Figure 6. Considering the gestational age in CCHD, preterm and full-term pregnancies were evenly divided. Preterm instances made up only 22.2% of PPHN patients, with the majority (77.8%) being full-term cases. Preterm patients (65.4%) had a higher prevalence of RDS than full-term cases (34.6%).

In CCHD, there was no significant difference in the mode of delivery between cesarean and natural. In contrast, PPHN and RDS display a preponderance of cesarean births (77.8% and 80.8%, respectively), compared to natural births (22.2% and 19.2%, respectively).

Both lower and normal birth weight cases of PPHN had a somewhat balanced distribution. Three CCHD cases had lower birth weights, compared to one with a normal birth weight. A total of 34.6% of RDS cases had normal birth weights, while 65.4% had lower birth weights. Given the distribution of genders, male children were more likely to have CCHD, RDS, and PPHN. The distribution of hemoglobin was balanced between levels below 14 and 14-18 g/dl.

**Figure 7:**
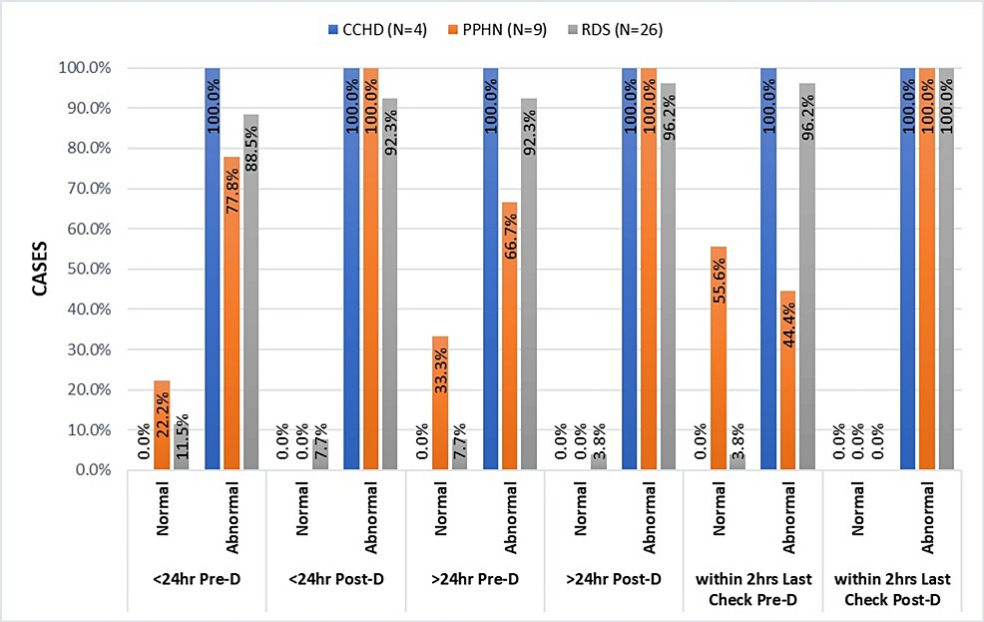
Comparative analysis of SpO2 levels in CCHD, RDS, and PPHN CCHD, critical congenital heart disease; PPHN, persistent pulmonary hypertension; RDS, respiratory distress syndrome

Regardless of the condition, it is evident from the bar graph analysis (Figure 7) that there were more abnormal post-ductal SpO2 readings than pre-ductal.

Throughout the study, all neonates with a CCHD diagnosis exhibited only abnormal readings, with SpO2 values ranging from 83% to 94%. Throughout the timeline, there was an increase in the number of normal pre-ductal SpO2 values in the babies with PPHN. The pre-ductal and post-ductal readings showed a 3-7% difference. With each screening test, the percentage of normal pre-ductal SpO2 readings decreased for neonates with RDS, with values often <90%.

**Figure 8:**
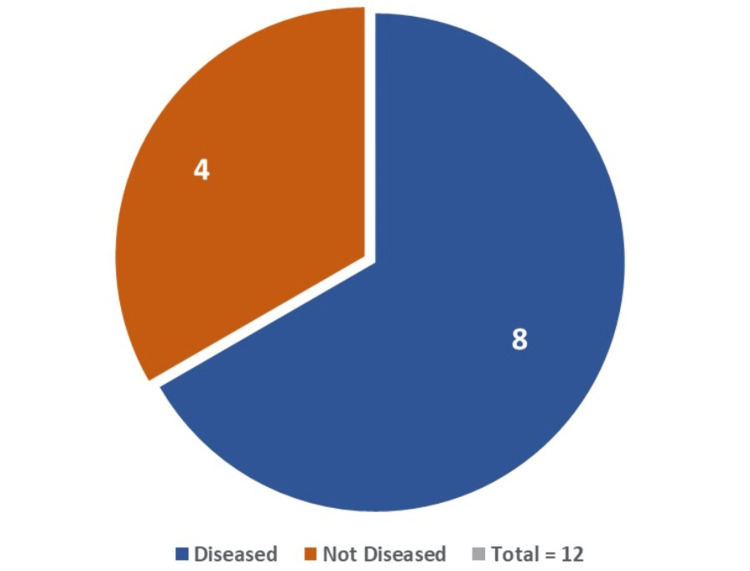
Diseased and non-diseased neonates with >3% difference in pre-ductal and post-ductal SpO2 CCHD = one case; PPHN = six cases; RDS = one case CCHD, critical congenital heart disease; PPHN, persistent pulmonary hypertension; RDS, respiratory distress syndrome

Of the 12 cases involving newborns with a difference of >3% between pre-ductal and post-ductal SpO2, eight cases were found to have a disease (CCHD = one case, PPHN = six cases, and RDS = one case) (Figure 8).

## Discussion

In India and many other nations, including the USA, China, and several European countries, pulse oximetry is routinely used for screening neonates for CCHD [23-25]. A pulse oximeter is more than just a screening tool; it is also used to evaluate how well disorders like RDS and PPHN are responding to treatment [26,27].

By the end of the study, 440 neonates had undergone POS tests before and after 24 hours. Not a single baby was examined precisely at 24 hours. As seen in Table 1, the study population exhibits a near-even gender distribution of males (52.5%) and females (47.5%), with natural deliveries (60.9%) being more prevalent than cesarean deliveries (39.1%). The majority of infants weigh between 2.5 and 4 kg with a median weight of 2.8 kg, and the distribution of hemoglobin levels and gestational ages provides a nuanced understanding of the health profile of the participants.

After thoroughly reviewing Table 2, it is evident that the majority of cases exhibit normal SpO2 levels (≥95%), with more cases on the POS test >24 hours than <24 hours after birth. Data reveals neonates have a greater number of normal pre-ductal values than post-ductal SpO2 values. This emphasizes the prevalence of normal oxygen saturation in the given population, irrespective of ductal location or time after the last test. A similar finding was seen in a study done by Said Habib [28].

In the category of SpO2 levels between 90 and 94% (Table 2), the distribution is roughly comparable, consisting of about 10.7-13% of the given population. The data reveals a similar pattern across different time points, except that there are more post-ductal abnormal SpO2 values as compared to the pre-ductal. For SpO2 levels below 90%, the distribution is consistent across all time intervals and ductal locations, with about 2.7-4.3% recorded cases in the given population. Throughout the screening period, SpO2 values were below 90% in two out of four CCHD, seven out of 26 RDS, and one out of nine PPHN patients. This data shows the same trend at various time points and pre-ductal and post-ductal locations. This suggests a persistent abnormality in oxygen saturation in these cases.

In reference to the SpO2 test performed on newborns showing abnormal results >24 hours after birth (Table 2), the screening test-positive cases again show a similar trend in the pre-ductal and post-ductal SpO2. It can also be seen that some cases show better SpO2 results post-test, making the screening test negative.

Analyzing Table 2 also reveals that there are more normal cases when testing occurs after 24 hours of birth, indicating reduced false positive screens when testing is done >24 hours after birth. Additionally, several other studies found that screening within 24-48 hours of birth reduces the number of false positives [23,29]. In summary, the table highlights the distribution of SpO2 levels in different scenarios, providing valuable insights into oxygen saturation patterns that may aid in clinical assessments and interventions. The consistent findings underscore the importance of monitoring and addressing variations in oxygen saturation to ensure optimal patient health.

Those with abnormal SpO2 readings before 24 hours followed by normal SpO2 readings during the second assessment after 24 hours were categorized as screening test negative. Nevertheless, they received ongoing monitoring and evaluation from doctors and nurses to ensure their well-being before being discharged. It is routine practice in our hospital to follow up on the neonate up to one week after discharge. Other neonates did not show any distress, and they were normal.

In India, the incidence of CHD is seven to nine per 1,000 live births. After echocardiography, four neonates out of 65 cases who tested positive for hypoxemia were found to have CCHD, yielding an incidence of 9.09% and a PPV of 6.15%. A similar study by Jain et al. reported a PPV of 12.2% [30]. Out of the four CCHD patients with diagnostic echocardiography, two had tetralogy of Fallot, one had transposition of great arteries, and the other had total anomalous pulmonary venous return.

Neonates diagnosed with CCHD and PPHN after echocardiography were promptly referred to higher medical centers for further treatment. In cases of mild to moderate RDS, symptoms typically manifest within 24 hours, often going unnoticed by parents, and peak around 48 hours. Our study’s timeframe of 48 hours ensured the safety of all neonates involved. When neonates consistently tested positive in our screening method, we promptly referred them for X-rays, echocardiography, and Silverman-Anderson scoring. Subsequently, they received immediate treatment, starting with admission to the NICU, followed by either humidified oxygen therapy or continuous positive airway pressure.

We utilized pulse oximetry as a screening tool for neonates with respiratory distress rather than for diagnostic purposes. The study excluded neonates who were extremely preterm (<32 weeks) and unstable at birth. Although the hospital advises a minimum stay of three to five days for neonates, some parents opt for early discharge. Once neonates are taken home, they start developing distress, and parents face limited options for treatment due to the scarce health resources in many Indian villages. Unfortunately, this has led to fatal outcomes for many neonates in India. Our test functions as a screening measure and does not replace the necessity of an X-ray for RDS diagnosis. Through our study, we were able to encourage parents to consider prolonging their hospital stay with compelling evidence of suboptimal SpO2 levels.

Despite the poor PPV for CCHD detection, the PPV for RDS and PPHN detection (PPV = 40% and 13.85%, respectively) by the POS test is relatively high, resulting in a combined PPV of 60% for the detection of CCHD and respiratory diseases. Because of this, the POS test is an excellent instrument for early detection and prompt referral for such severe illnesses. Some other studies also came to similar conclusions [8,30]. In addition to screening, pulse oximetry is an essential assessment for monitoring the prognosis of newborns receiving care for any of these conditions [20,22].

Strengths

The Masimo Rad-67® Pulse CO-Oximeter’s precision and portability are two key advantages for this research project. Although there may not be a portable pulse oximeter available in rural areas, traditional pulse oximeters used in rural community healthcare centers also produce equivalent results. In India, pulse oximeters come in a range of brands and are reasonably priced [30]. Additionally, this study offers a comparison of the POS values obtained less than 24 hours and more than 24 hours after birth.

Limitations

The study’s brief duration and the fact that medical students are conducting it under the supervision of an MD pediatrics professor mean that there are slightly fewer cases than expected. Not all research participants could have an echocardiogram performed to evaluate false negatives and true negatives due to resource limitations. Therefore, we are unable to remark on the test’s negative predictive value, specificity, or sensitivity. As the research is carried out in a remote community health center, the findings are limited to other rural populations with comparable healthcare environments.

## Conclusions

This in-depth analysis highlights the distinctive characteristics and trends associated with each neonatal condition within the study population, providing valuable insights for clinical understanding and management. These findings serve as a baseline for further research, contribute to our knowledge of neonatal health, and inform clinical approaches for these conditions.

Our study emphasizes the significance of conducting routine POS in conjunction with appropriate clinical examination in low-resource rural healthcare settings to identify and refer cases of CCHD, PPHN, and RDS early on.

## References

[1] Jubran A (2015). Pulse oximetry. Crit Care.

[2] Prudhoe S, Abu-Harb M, Richmond S, Wren C (2013). Neonatal screening for critical cardiovascular anomalies using pulse oximetry. Arch Dis Child Fetal Neonatal Ed.

[3] Phillipos E, Solevåg AL, Aziz K (2017). Oxygen saturation and heart rate ranges in very preterm infants requiring respiratory support at birth. J Pediatr.

[4] Sweet DG, Carnielli V, Greisen G (2019). European Consensus Guidelines on the management of respiratory distress syndrome - 2019 update. Neonatology.

[5] (2020). Global, regional, and national burden of congenital heart disease, 1990-2017: a systematic analysis for the Global Burden of Disease Study 2017. Lancet Child Adolesc Health.

[6] Bernier PL, Stefanescu A, Samoukovic G, Tchervenkov CI (2010). The challenge of congenital heart disease worldwide: epidemiologic and demographic facts. Semin Thorac Cardiovasc Surg Pediatr Card Surg Annu.

[7] van der Linde D, Konings EE, Slager MA, Witsenburg M, Helbing WA, Takkenberg JJ, Roos-Hesselink JW (2011). Birth prevalence of congenital heart disease worldwide: a systematic review and meta-analysis. J Am Coll Cardiol.

[8] Plana MN, Zamora J, Suresh G, Fernandez-Pineda L, Thangaratinam S, Ewer AK (2018). Pulse oximetry screening for critical congenital heart defects. Cochrane Database Syst Rev.

[9] Lozano R, Naghavi M, Foreman K (2012). Global and regional mortality from 235 causes of death for 20 age groups in 1990 and 2010: a systematic analysis for the Global Burden of Disease Study 2010. Lancet.

[10] Oster ME, Lee KA, Honein MA, Riehle-Colarusso T, Shin M, Correa A (2013). Temporal trends in survival among infants with critical congenital heart defects. Pediatrics.

[11] Brown KL, Ridout DA, Hoskote A, Verhulst L, Ricci M, Bull C (2006). Delayed diagnosis of congenital heart disease worsens preoperative condition and outcome of surgery in neonates. Heart.

[12] Fixler DE, Xu P, Nembhard WN, Ethen MK, Canfield MA (2014). Age at referral and mortality from critical congenital heart disease. Pediatrics.

[13] Wren C, Reinhardt Z, Khawaja K (2008). Twenty-year trends in diagnosis of life-threatening neonatal cardiovascular malformations. Arch Dis Child Fetal Neonatal Ed.

[14] Abu-Harb M, Hey E, Wren C (1994). Death in infancy from unrecognised congenital heart disease. Arch Dis Child.

[15] (2024). NCHDA 2019 summary report. https://www.hqip.org.uk/wp-content/uploads/2019/09/Ref-129-Cardiac-NCHDA-Summary-Report-2019-FINAL.pdf.

[16] van Velzen CL, Ket JC, van de Ven PM, Blom NA, Haak MC (2018). Systematic review and meta-analysis of the performance of second-trimester screening for prenatal detection of congenital heart defects. Int J Gynaecol Obstet.

[17] Singh A, Rasiah SV, Ewer AK (2014). The impact of routine predischarge pulse oximetry screening in a regional neonatal unit. Arch Dis Child Fetal Neonatal Ed.

[18] de-Wahl Granelli A, Wennergren M, Sandberg K (2009). Impact of pulse oximetry screening on the detection of duct dependent congenital heart disease: a Swedish prospective screening study in 39,821 newborns. BMJ.

[19] Ewer AK, Middleton LJ, Furmston AT (2011). Pulse oximetry screening for congenital heart defects in newborn infants (PulseOx): a test accuracy study. Lancet Lond Engl.

[20] Sweeney EL, Kallapur SG, Gisslen T (2016). Placental infection with ureaplasma species is associated with histologic chorioamnionitis and adverse outcomes in moderately preterm and late-preterm infants. J Infect Dis.

[21] Steinhorn RH (2013). Diagnosis and treatment of pulmonary hypertension in infancy. Early Hum Dev.

[22] Walsh-Sukys MC, Tyson JE, Wright LL (2000). Persistent pulmonary hypertension of the newborn in the era before nitric oxide: practice variation and outcomes. Pediatrics.

[23] Garne E, Stoll C, Clementi M (2001). Evaluation of prenatal diagnosis of congenital heart diseases by ultrasound: experience from 20 European registries. Ultrasound Obstet Gynecol.

[24] Huang Y, Zhong S, Zhang X (2022). Large scale application of pulse oximeter and auscultation in screening of neonatal congenital heart disease. BMC Pediatr.

[25] Klausner R, Shapiro ED, Elder RW, Colson E, Loyal J (2017). Evaluation of a screening program to detect critical congenital heart defects in newborns. Hosp Pediatr.

[26] Gopalakrishnan S, Karmani S, Pandey A, Singh N, Ratheesh Kumar J, Praveen R, Sodhi K (2021). Pulse oximetry screening to detect critical congenital heart diseases in asymptomatic neonates. Med J Armed Forces India.

[27] Wick KD, Matthay MA, Ware LB (2022). Pulse oximetry for the diagnosis and management of acute respiratory distress syndrome. Lancet Respir Med.

[28] Said Habib H (2013). Oxygen saturation trends in the first hour of life in healthy full-term neonates born at moderate altitude. Pak J Med Sci.

[29] Riede FT, Wörner C, Dähnert I, Möckel A, Kostelka M, Schneider P (2010). Effectiveness of neonatal pulse oximetry screening for detection of critical congenital heart disease in daily clinical routine—results from a prospective multicenter study. Eur J Pediatr.

[30] Jain D, Jain M, Lamture Y (2022). Pulse oximetry screening for detecting critical congenital heart disease in neonates. Cureus.

